# Adiponectin, resistin, Interleukine-4, TGF-β2 levels before and after clozapine treatment in a group of first episode, treatment resistant schizophrenia patients

**DOI:** 10.1192/j.eurpsy.2023.1326

**Published:** 2023-07-19

**Authors:** P. Petrikis, A. Karampas, G. Leondaritis, D. Archimandriti, P. Spyrou, M. Plakoutsis, G. Georgiou, C. Mantas, P. Voulgari, T. Hyphantis

**Affiliations:** 1 Department of psychiatry, university of ioannina-faculty of medicine, Ioannina-Greece; 2 Department of psychiatry; 3 Department of pharmacology; 4 Department of rheumatology-immunology; 5 Department of eheumatology-immunology, university of ioannina-faculty of medicine, Ioannina, Greece

## Abstract

**Introduction:**

Treatment resistant schizophrenia (TRS) affects at least 15-20% of newly diagnosed patients with schizophrenia. Clozapine treatment for this sub-group of patients should be considered immediately after the failure in response to two antipsychotics (usually risperidone and olanzapine) received in adequate doses and time. Immune changes have been reported in first episode patients with psychosis, although results from various studies remain inconclusive. Very few studies have investigated so far cytokine and adipokine changes in TRS.

**Objectives:**

To measure Interleukin-4 (IL-4), Tumor Growth Factor-β2 (TGF-β2), adiponectin and resistin in a sample of forty first-episode patients with psychosis (FEP) who proved to be treatment resistant (TRS),after treatment with two atypical antipsychotics ( olanzapine and risperidone) and after clozapine treatment.

**Methods:**

Serum levels of adiponectin, resistin, IL-4 and TGF-β2 were measured by enzyme linked immunosorbent assay (ELISA) before and after clozapine treatment. Psychopathology was assessed with the Positive and Negative Syndrome Scale (PANSS) before and after clozapine treatment, BMI was calculated in the days of blood sample collection.

**Results:**

We observed a significant decrease of adiponectin levels after switching from olanzapine/risperidone to clozapine (p<0.0001, Wilcoxon signed rank test W=820.0) and a statistically significant increase of resistin levels after switching to clozapine (p<0.0001, Wicoxon signed rank test W=-658.0). Similarly, a significant increase of TGF-β2 and of IL-4 levels was found after clozapine treatment (p=0.039, Wilcoxon signed rank test test W=-454.0 and p=0.029, Wilcoxon signed rank test W=-386.0, respectively).

**Conclusions:**

Resistin, IL-4 and TGF-β levels increase after clozapine treatment while adiponectin levels significantly decrease. The findings may of importance, since they may reflect specific immune changes to clozapine responders.

**Disclosure of Interest:**

None Declared

